# Testing the Effectiveness of Enhanced Alcohol Warning Labels and Modifications Resulting From Alcohol Industry Interference in Yukon, Canada: Protocol for a Quasi-Experimental Study

**DOI:** 10.2196/16320

**Published:** 2020-01-10

**Authors:** Kate Vallance, Timothy Stockwell, David Hammond, Simran Shokar, Nour Schoueri-Mychasiw, Thomas Greenfield, Jonathan McGavock, Jinhui Zhao, Ashini Weerasinghe, Erin Hobin

**Affiliations:** 1 Canadian Institute for Substance Use Research University of Victoria Victoria, BC Canada; 2 School of Public Health & Health Systems University of Waterloo Waterloo, ON Canada; 3 Canadian Agency for Drugs and Technologies in Health Toronto, ON Canada; 4 Health Promotion, Chronic Disease and Injury Prevention Public Health Ontario Toronto, ON Canada; 5 Alcohol Research Group Public Health Institute Emeryville, CA United States; 6 Department of Pediatrics and Child Health Faculty of Health Sciences University of Manitoba Winnipeg, MB Canada

**Keywords:** alcohol warning labels, alcohol policy, alcohol, cancer, national drinking guidelines, standard drink labels, alcohol industry

## Abstract

**Background:**

Alcohol warning labels are a promising, well-targeted strategy to increase public awareness of alcohol-related health risks and support more informed and safer use. However, evidence of their effectiveness in real-world settings remains limited and inconclusive.

**Objective:**

This paper presents a protocol for a real-world study examining the population-level impact of enhanced alcohol warning labels with a cancer message; national drinking guidelines; and standard drink information on attention, processing, and alcohol-related behaviors among consumers in Canada. Postimplementation modifications to the original protocol due to interference by national alcohol industry representatives are also described.

**Methods:**

This quasi-experimental study involved partnering with local governments in two northern Canadian territories already applying alcohol warning labels on alcohol containers for sale in liquor stores. The study tested an 8-month intervention consisting of three new enhanced, rotating alcohol warning labels in an intervention site (Whitehorse, Yukon) relative to a comparison site (Yellowknife, Northwest Territories) where labelling practices would remain unchanged. Pre-post surveys were conducted at both sites to measure changes in awareness and processing of label messages, alcohol-related knowledge, and behaviors. Liquor store transaction data were collected from both sites to assess changes in population-level alcohol consumption. The intervention was successfully implemented for 1 month before it was halted due to complaints from the alcohol industry. The government of the intervention site allowed the study to proceed after a 2-month pause, on the condition that the cancer warning label was removed from rotation. Modifications to the protocol included applying the two remaining enhanced labels for the balance of the intervention and adding a third wave of surveys during the 2-month pause to capture any impact of the cancer label.

**Results:**

This study protocol describes a real-world quasi-experimental study that aimed to test the effectiveness of new enhanced alcohol warning labels as a tool to support consumers in making more informed and safer alcohol choices. Alcohol industry interference shortly after implementation compromised both the intervention and the original study design; however, the study design was modified to enable completion of three waves of surveys with cohort participants (n=2049) and meet the study aims.

**Conclusions:**

Findings from this study will directly inform alcohol labelling policies in Canada and internationally and provide further insight into the alcohol industry’s attempts to disrupt research in this area. Additional unimpeded real-world evaluations of enhanced alcohol warning labels are recommended.

**International Registered Report Identifier (IRRID):**

RR1-10.2196/16320

## Introduction

Alcohol poses significant public health and safety problems in Canada, which are especially severe in its northern territories [1,2]. Harms and economic costs of alcohol consumption are increasing nationally and were recently estimated at CAD $14.6 billion per annum, greater than all other psychoactive substances including tobacco. Per capita alcohol costs were at least double the national average in the north. In 2016, alcohol was responsible for 14,800 deaths (including 4275 from cancer), 87,000 hospital admissions, and 139,000 productive years of life lost in Canada [2]. Despite the extent of this harm, there are low levels of awareness of health risks from alcohol, particularly cancer risk [3], and of national low-risk drinking guidelines (LRDG) among Canadians [4-6].

Alcohol warning labels are a promising strategy for increasing public awareness of alcohol-related health risks and supporting safer consumption [7,8]. They are a unique intervention providing critical information both when alcohol is purchased and during consumer use. Alcohol warning labels also have a broad reach, as nearly all drinkers are exposed to labels; those who consume alcohol more heavily are exposed more often and are thus most likely to recall these messages [9,10]. Based on studies of tobacco warning labels, key label elements include a relatively large size and font, full color graphics or images, personally relevant and direct messages, and prominent placement on packages [11-13]. Implementing rotating messages to prevent a wear-out effect over time and having accompanying education campaigns also increase the effectiveness of warning labels in the context of tobacco and alcohol [11,14].

Alcohol warning labels are currently mandated on alcohol containers in 47 countries worldwide, although not in Canada—with the exception of two northern jurisdictions applying labels by local directives. However, to date, labels used internationally are small, are not prominently displayed on containers, and virtually all contain text-only messages with vague statements and minimal graphics [15,16]. There is increasing evidence of concerted efforts by the global alcohol industry to embed alcohol in the fabric of society and minimize or misrepresent information presented to the public on alcohol-related harms [17,18], suggesting that the existence of such few instances of labels following best practice guidelines may not be accidental. In 2010, Thailand was set to introduce a series of labels that included graphic images representing alcohol harms in a similar style to those used successfully on tobacco packages. However, significant push back from trade organizations representing major alcohol-producing countries prevented the labels from being implemented [19]. More recently, alcohol suppliers in the United Kingdom negotiated a private deal with the government, allowing an additional 30-month “grace period” before commencing implementation of mandatory health messaging on alcohol products, a requirement introduced in 2016, and which included listing newly lowered national drinking guidelines; the public was only informed of the deal 22 months into the grace period and the outdated higher drinking guidelines still remain on many products [20].

The majority of evaluations that have tested the effectiveness of existing alcohol warning labels have focused on the mandatory label introduced in the United States in 1989 [9,21,22]. The US label, which states that drinking alcohol while pregnant can cause birth defects and that consumption of alcohol impairs ability to drive a car and operate machinery and may cause health problems, is text-only with no requirements for color, message rotation, or prominent placement on alcohol containers. Not surprisingly, effects of this label were limited mainly to situational preventive behaviors (not drinking before driving, not driving after drinking), sparking conversations about drinking and pregnancy and interventions by collaterals to prevent someone else from drunk driving, with the heaviest drinkers reporting the highest recall of label messages [9,22]. Lab-based and smaller-scale qualitative studies conducted in Canada and elsewhere revealed that consumers were open to prominently displayed alcohol warning labels that included a health message such as a cancer warning [23-25], standard drink information, and LRDG [6,23,25]. When tested in Canada, the drinking guidelines and standard drink information helped consumers more accurately calculate the number of standard drinks in a bottle and better track their consumption in relation to the LRDG [6,23].

There is a dearth of evidence measuring the effectiveness of enhanced alcohol warning labels that follow best practice recommendations, especially those that include integral information for consumers such as alcohol-related harms including risk of cancer and information to facilitate safer drinking patterns. Accordingly, the primary objective of this study is to test, in a real-world setting, if labelling alcohol containers with a cancer message, standard drink information, and national drinking guidelines supports more informed and safer alcohol use and has a population-level impact on alcohol consumption. Specifically, our research questions include the following: (1) What is the effect of enhanced alcohol warning labels relative to usual labelling practice (comparison site) on noticing labels and recall of label messages; processing label messages; knowledge of alcohol-related health risks, national drinking guidelines, and the concept of a standard drink; and self-reported drinking behaviors? (2) What is the population-level effect of enhanced alcohol warning labels on alcohol consumption relative to usual labelling practice (comparison site)? In this paper, we present the original protocol of this controlled pre-post quasi-experimental study and describe how the study design was modified postimplementation in response to efforts by representatives of the national alcohol industry in Canada to disrupt the study.

## Methods

### Setting

**Figure 1:**
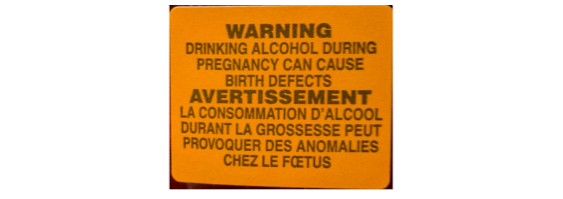
Alcohol warning label on alcohol containers in Yukon prior to the intervention (2.3 cm x 2.8 cm).

**Figure 2:**
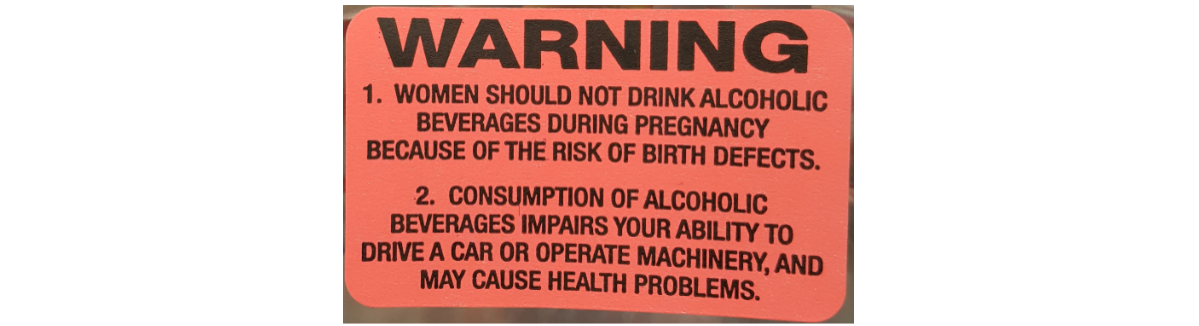
Alcohol warning label on alcohol containers in the Northwest Territories prior to and during the intervention (3.0 cm x 5.0 cm).

This study was conducted in two capital cities (Whitehorse, Yukon, and Yellowknife, Northwest Territories) located in two of Canada’s three vast and sparsely populated northern territories. Whitehorse (population=28,225 as of 2016) and Yellowknife (population=19,569 as of 2016) are appropriate matched sites for a number of reasons: They have similar government-run alcohol distribution systems that account for almost all off-premise alcohol sales in both cities and comparable per capita alcohol sales, population size, ethnic diversity, age, education levels, and income profiles [2,26-29]. Whitehorse has one mid-sized government-run liquor store and a handful of private stores or licensed premises such as bars or hotels that can sell off-sales; there are five small government-run agency stores located in five small communities across Yukon. Yellowknife has limited off-sales available from licensed premises and two government-run agency stores; there are five small agency stores located in five small communities across the rest of the territory. Further, both territories have had exposure to alcohol warning labels since 1991 when application of a postmanufacture label on containers cautioning consumers about drinking while pregnant (with an additional warning about alcohol and driving and general health risks in Northwest Territories) became mandatory by territorial directives (Figures 1 and 2). No other jurisdictions in Canada currently require alcohol warning labels.

During the development of the study, the research team approached provinces and territories with an invitation to participate in the labelling experiment. The government-run Yukon Liquor Corporation, responsible for the sale and distribution of alcohol in the territory, accepted and agreed to participate in the study as the intervention site. The single flagship liquor store in Whitehorse presented an ideal and practical intervention site and offered maximum exposure to the new labels with well-established labelling procedures already in place. The Northwest Territories’ government-run Liquor Commission agreed to act as a comparison site, which involved making no changes to labelling practices at their two Yellowknife retail liquor stores during the intervention period.

### Ethics Approval and Consent to Participate

This study received ethics approval from the Research Ethics Board at Public Health Ontario (identification number: 2017-010.04) and the Human Research Ethics Board at the University of Victoria (Protocol 17-161) and obtained the relevant research licenses required in Yukon and Northwest Territories. Written, informed consent was obtained from all study participants prior to completing the survey, and participants were also provided with a letter of information about the study.

### Alcohol Warning Label Intervention

**Figure 3:**
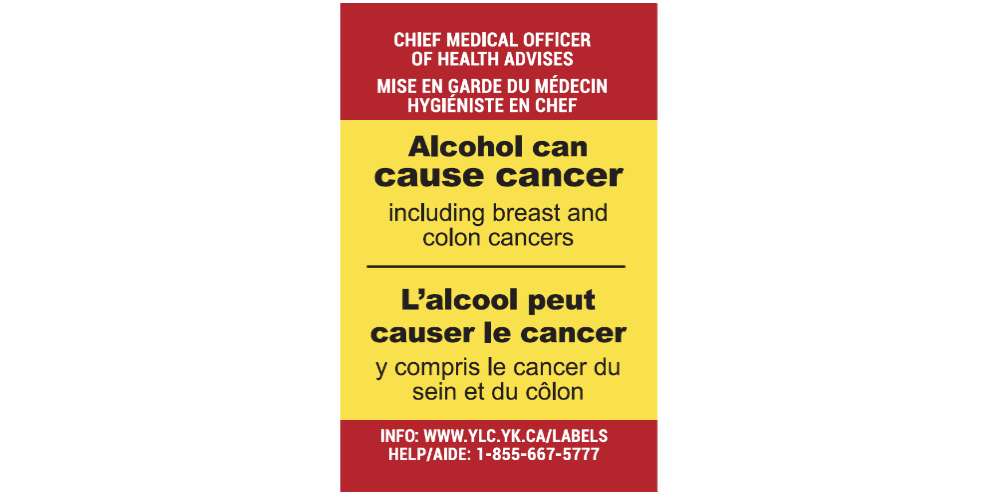
Intervention alcohol warning label: Cancer warning (5.0 cm x 3.2 cm).

**Figure 4:**
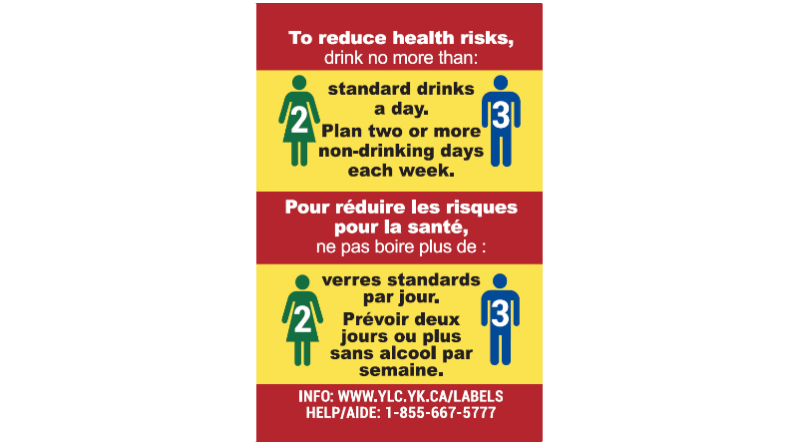
Intervention alcohol warning label: Low-risk drinking guidelines (5.0 cm x 3.2 cm).

**Figure 5:**
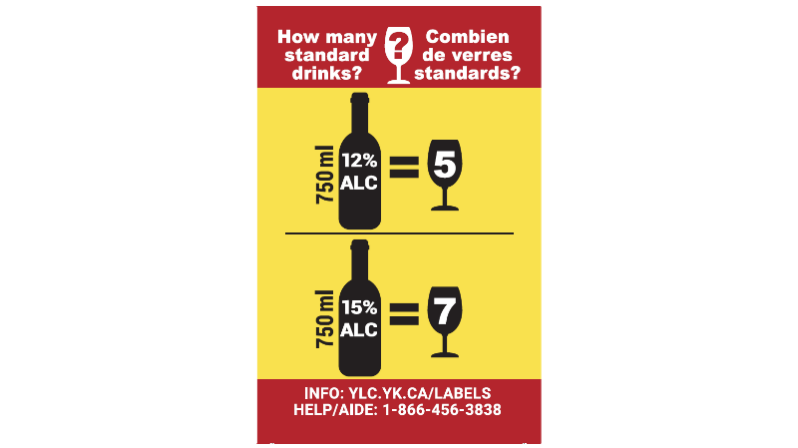
Intervention alcohol warning label: Standard drink information - example for 750 mL wine (5.0 cm x 3.2 cm).

The labelling intervention involved rotating three new evidence-informed enhanced alcohol warning labels (Figures 3-5) on all alcohol containers (with the exception of select local and single-serve beer and single-serve cider, ~3% of total sales) in the intervention site liquor store for an 8-month period. Intervention labels were applied in store by trained liquor store staff, using specialized label-application guns. Consistent with evidence for effective labelling initiatives [11,14,30], a social marketing and awareness campaign including in-store signage, handouts, a website, toll-free helpline, and radio spots that incorporated the three label messages was planned to run parallel to the timeframe of the intervention.

The design of the enhanced intervention labels was directly informed by the results of a between-group experiment testing the efficacy of the label content, format and size [23], and focus groups among residents across Yukon to further refine the label design and gauge consumer acceptability of the labels [25]. The labels were also reviewed during extensive consultations with international experts and local public health and community stakeholders in Yukon. As per territorial government regulations, all label messaging was required to be presented in both English and French, Canada’s two official languages. To address resulting label-size considerations, the final messages were presented on three separate rotating labels.

As shown in Figures 3-5, the three new enhanced alcohol warning labels improved on the existing label in the intervention site in a number of ways: They were larger in size, used a bright yellow background and red border, and incorporated graphics along with the three distinct health messages. The first label (Figure 3) stated that alcohol can cause cancer, which is an evidence-based statement [31-37], specifically mentioning breast and colon cancers, two prevalent cancers in Canada [1,38]. The second label (Figure 4) presented Canada’s LRDG for men and women [39] using an infographic, and the third label (Figure 5), also using an infographic, provided consumer information on how many standard drinks are contained in different size and strength alcoholic beverages [40]. Standard drink labels with the most common alcohol strengths were developed for standard size wine (750 mL, 12% and 15%), spirits (750 mL, 40% and 60%), beer (355 mL, 5% and 7.5%) and cider (2 L, 7%) containers; a standard drink in Canada contains 13.45 mg of pure alcohol.

### Study Design and Data Sources

**Figure 6:**
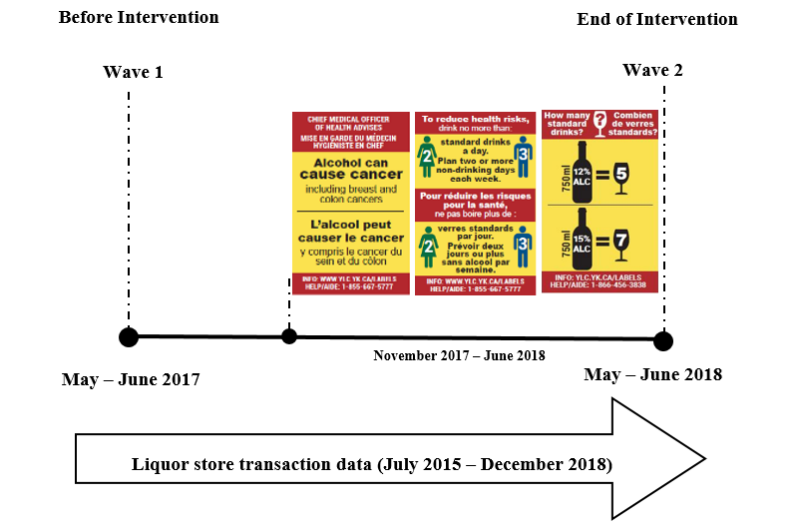
Original study design and timeline.

A pre-post quasi-experimental study with the comparison site was designed to test the impact of the 8-month labelling intervention. No randomization was applied as full voluntary cooperation, and participation of the intervention site was required for implementation of the alcohol warning labels intervention. The study included two data sources from both the intervention and comparison sites: (1) surveys with cohort participants, and (2) aggregated liquor store-level sales data (Figure 6). Pre-post surveys included a split-panel design with a panel cohort nested within two repeated cross-sectional data collections to assess participant responses. Wave 1 surveys were conducted in the intervention and comparison sites in May/June 2017, 4 months before the label intervention was to be implemented in the intervention site in November 2017. Wave 2 surveys were scheduled for both sites in May/June 2018, 8 months after the label intervention was implemented in the intervention site. Contact information provided by participants at Wave 1 allowed for email recruitment at Wave 2 using a time-limited online survey. All survey periods continued for 6 weeks, the survey was approximately 18 minutes in length, survey measures were consistent across waves and sites, and participants received a gift card to a national coffee store chain or an Interac electronic transfer as remuneration in appreciation of their time.

Arranged by prior agreement with governments in both the intervention and comparison sites, aggregated product-level liquor store sales data spanning the months before, during, and after implementation of the intervention labels were also obtained. Analyses of sales data were designed to estimate the impact of the labels on population-level alcohol consumption, specifically the total volume, type, and strength of alcohol purchases in liquor stores before, during, and after the intervention. Alcohol sales data are a robust and standard measure for tracking target population alcohol intake over time and for cross-jurisdictional comparisons, as purchases are predictive of consumption [41].

### Surveys

#### Survey Recruitment and Procedures

In Wave 1, surveys were conducted with liquor store customers in the intervention and comparison sites over a 6-week period from May to June 2017. Trained research assistants were stationed in liquor store lobbies from Monday to Saturday in both sites; all stores were closed on Sundays. Recruitment in the intervention site took place each day during opening hours of the store between 10 AM to 6 PM and during extended Friday opening hours between 10 AM to 8 PM. Store opening hours in the comparison site were between 11 AM to 11 PM each day, and recruitment took place between 12 PM and 8 PM; recruitment hours in the comparison site were carefully selected to cover peak sale periods equivalent to those in the intervention site. Customers were systematically selected to participate in the survey upon exiting the liquor store using a standard intercept technique of approaching every person that passed a preidentified landmark in the liquor store. Eligibility for the survey was established through a screening tool: Participants had to be aged ≥19 years (the legal drinking age in both sites), have consumed at least one alcoholic drink in the past month, be residents of the intervention or comparison site cities, not currently self-report being pregnant or breastfeeding, and have purchased liquor at the store that day. Eligible participants were required to read and sign consent forms prior to starting the survey. The survey was completed independently in English on a 10-inch tablet with no interviewer assistance; the majority of the population was English speaking at both sites. Because the main objectives of the study were to assess the extent to which consumers notice, understand, and use alcohol warning labels when purchasing and consuming alcohol, per agreement of the human subjects committees, the purpose of the study was only partially disclosed to the participants to avoid influencing participant responses.

In May 2017, a small feasibility study was conducted with 20 participants at the liquor store at the intervention site. The study tested all aspects of the survey including recruitment, measures, and data collection protocol. Prior to the feasibility study, cognitive interviewing was conducted with a convenience sample of participants as a pretest to assess survey interpretation and comprehension.

#### Survey Measures

Survey measures were adapted from those used in previous evaluations of the alcohol warning labels used in the United States, of Canadian food labelling systems, and of tobacco warning labels to collect data on both the existing and new enhanced alcohol warning labels [13,24,25,27-29,42,43]. Select primary and secondary outcomes are listed in Table 1.

**Table 1:** Select primary and secondary outcomes.

Outcomes and question			Response options
**Primary**			
	**Noticing labels**		
		“In the past 6 months, have you seen any warning labels on bottles or cans of beer, wine, hard liquor, coolers, or ciders?”	No/Yes/Don’t know/Prefer not to say
	**Label recall**		
		“In the past 6 months, what messages have you seen on the warning labels on bottles or cans of beer, wine, hard liquor, coolers, or ciders? (Please list all messages you have seen on labels.)”	Open-ended text field/Don’t know/Prefer not to say
	**Label processing**		
		“In the past 6 months, how often, if at all, have you read or looked closely at/thought about/talked about with others the warning labels on bottles or cans of beer, wine, hard liquor, coolers, or ciders?”	Never/Rarely/Sometimes/Often/Very often/Don’t know/Prefer not to say
	**Self-reported behavior change due to labels**		
		“In the past 6 months, has the amount of alcohol you are drinking changed as a result of the warning labels on bottles or cans of beer, wine, hard liquor, coolers, or ciders? Are you drinking:	Less/Same amount/More/Don’t know/Prefer not say
	**Self-reported alcohol-drinking behaviors**		
		“During the past 6 months, how often did you drink alcoholic beverages?”	Less than once a month/Once a month/2 to 3 times a month/Once a week/2 to 3 times a week/4 to 6 times a week/Every day/Don’t know/Prefer not to say
		“During the past 6 months, on those days when you drank alcohol, how many drinks did you USUALLY have?”	Enter number of drinks: Open-ended numeric field/Don’t know/Prefer not to say
	**Sex-specific alcohol consumption on a single occasion**		
		“During the past 6 months, how often have you had 4 or more (if male participant)/3 or more (if female participant) drinks on one occasion?”	Never in the past 6 months/Less than once a month/Once a month/2 to 3 times a month/Once a week/2 to 5 times a week/Daily or almost daily/Don’t know/Prefer not to say
**Secondary**			
	**Alcohol-related health risks**		
		“Based on what you know or believe, can drinking alcohol cause breast cancer/liver disease/the flu/[when pregnant] harm to a fetus?”	No/Yes/Don’t know/Prefer not to say
	**Awareness of national drinking guidelines**		
		“Were you aware of Canada’s Low-Risk Drinking Guidelines before today?”	No/Yes/Don’t know/Prefer not to say
	**Knowledge of standard drink measurements**		
		“How many ‘standard drinks’ are in this bottle of [preferred drink type]?” (image of their preferred drink type shown on tablet screen)	Enter number of drinks: Open numerical field/Don’t know/Prefer not to say
	**Self-reported use of label information**		
		“If the number of standard drink were displayed on bottles and cans of alcoholic drinks, like the one shown on the screen, would you ever use this information to help yourself or someone else stay within the daily drink limit advised by in the low-risk drinking guidelines?”	No/Yes/Don’t know/Prefer not to say
		“To what extent, if at all, would labels with low-risk drinking guidelines on bottles and cans of alcoholic beverages make you think about the number of drinks you consume?”	Not at all/Not much/Neutral/Somewhat/Very much/Don’t know/Prefer not to say
	**Support for alcohol labelling and other alcohol-related policies**		
		“Cans and bottles of alcoholic beverages should be labelled with low-risk drinking guidelines/the number of standard drinks per container/warnings describing the link between alcohol and diseases, such as cancer.”	Strongly disagree/Disagree/Neutral/Agree/Strongly agree/Don’t know/Prefer not to say

Participants were informed that a “drink” refers to 341 mL of beer, 5 oz of wine, and 1.5 oz of spirits. Sociodemographic variables were assessed using survey items adapted from national surveys in Canada, and health literacy was assessed by the validated 6-item New Vital Sign measure [4,44]. Additional response options including *Don’t know* and *Prefer not to say* were also presented across all survey questions.

#### Sample Size Calculation

Target sample sizes of 406 per condition per wave was calculated prior to the study to provide 80% power to detect a minimum difference of 8% in the proportion of “Yes” or correct responses to the cognitive processing outcomes with a two-tailed test, where *a*=.05. The final sample size was inflated by 10% up to 450 participants per condition to account for missing data on key measures. Estimates were based on data from the evaluation of the alcohol warning labels used in the United States [9].

#### Survey Analyses

Similar to previous evaluations of label interventions, variables such as awareness and recall of label messages and knowledge of related health risks were assessed as binary outcomes (0=No/Don’t know vs 1=Yes). Volume of alcohol consumption was calculated as the mean number of drinks consumed per week in the past 6 months. For baseline (Wave 1) data, logistic regression analyses were performed to examine the relationship between select primary and secondary outcome measures and sociodemographic factors; adjusted odds ratio and corresponding 95% CI of the outcomes for sociodemographic factors was estimated [45]. Generalized estimating equation models using a binomial distribution with logit link function were used to examine the longitudinal effects of the alcohol warning labels intervention on all secondary outcome measures across the three waves. Generalized estimating equation models can account for a mix of within-subject correlation that arises from the cohort participants being asked the same questions over multiple survey waves plus the replenishment sample [46]. The large overlap in the cohort sample across waves implies that observations were not and cannot be treated as independent. Difference-in-difference terms with an interaction between wave and site were added to each model to assess the changes in outcomes across survey waves and between sites. Sociodemographic variables and other covariates, such as time in sample, were also included in all models. All analyses were conducted using SAS 9.3 (SAS Institute Inc, Cary, North Carolina).

### Liquor Store Sales Data

#### Sales Data

Product-level liquor store sales data, provided by the Yukon government alcohol monopoly, included all retail and wholesale purchases in the Whitehorse liquor store and the five liquor stores in the surrounding areas (Dawson, Faro, Haines Junction, Mayo, and Watson Lake) from July 2015 to December 2018. These data were aggregated by units and volume sizes; beverage category (beer, wine, spirits, coolers, and cider); percent alcohol/volume (eg, <4%, 4%-5.4%, 5.5%-6.9%, ≥7% for beer); site; and month. Liquor store sales data provided data at the store level and did not include individual-level customer data or financial information. Overall, this provided 28-month baseline and 14-month follow-up data, enabling estimation of seasonal effects and secular sales trends for control in analyses. Multilevel regression analyses of pooled six time series alcohol data also controlled for regional effects. The Northwest Territories’ sales data were included in the analysis as an additional control. Total monthly alcohol sales for the Northwest Territories, including the two liquor stores in Yellowknife, were retrieved from the Northwest Territories Bureau of Statistics for the same time period [47]. Per capita alcohol consumption was estimated for individuals aged ≥15 years in Yukon and Northwest Territories for each monthly period by dividing the total liters of ethanol sold by the population aged ≥15 years as per data from Statistics Canada [24]. Dollar values were adjusted by consumer price index (CPI) using territory-specific CPI data from Statistics Canada [48].

#### Socioeconomic and Demographic Data

Several socioeconomic and demographic data by site and time period were obtained to produce per capita alcohol consumption estimates and socioeconomic variables in order to examine and control for their potential confounding effects [49-53]. These data included population data in Yukon [54] and Northwest Territories [55], income and CPI data [56-62], and land data [63].

#### Liquor Store Sales Data Analyses

Multilevel regression models [64] were used to analyze pooled monthly per capita retail alcohol sales in six areas (one single liquor store in Whitehorse, Dawson, Faro, Haines Junction, Mayo, and Watson Lake) in Yukon to examine the effect of alcohol warning labels on per capita alcohol consumption among people aged ≥15 years, adjusting for the potential confounding effects of annual household income, the proportion of young people aged 20-29 years, the proportion of men, the proportion of Indigenous populations, annual trends and seasonal variations, and temporal and regional autocorrelation. Separate models were created to examine specific effects of the labels by beverage type. Per capita alcohol consumption variables were log transformed to remove skewness in their distributions. The number of days in each month was used as a weighting variable; the number of days varied by month and the labels were initially applied starting mid-month. Estimates of monthly per capita alcohol consumption in the Northwest Territories based on official sales data for the same time period were used as an additional separate control in the models.

## Results

### Postimplementation Modifications to Study Design

**Figure 7:**
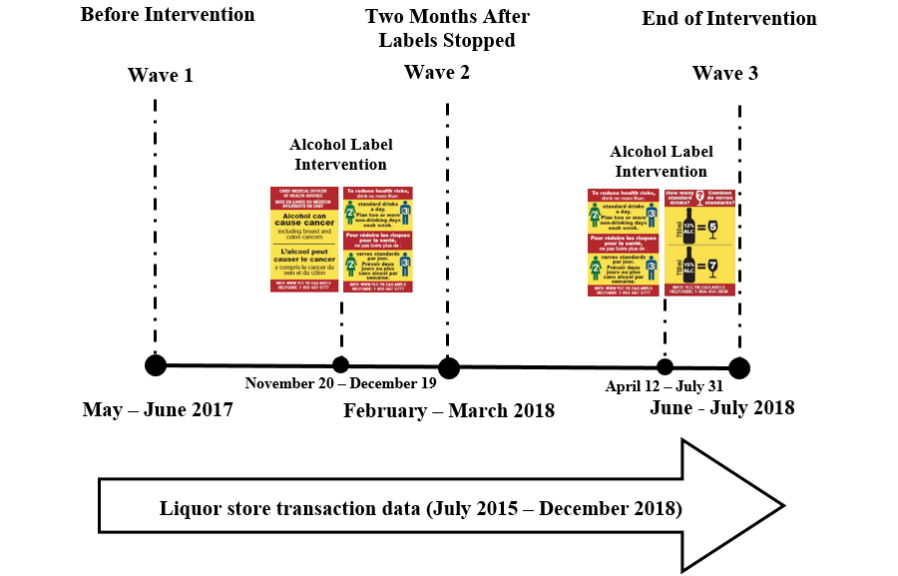
Modified study design and timeline.

Postimplementation modifications were made to the study design due to interference from Canadian alcohol industry trade associations. On December 19, 2017, 4 weeks after the intervention launched on November 20, the Yukon Liquor Corporation paused all application of the new enhanced labels for nearly 4 months due to complaints from the national industry representatives [65]. On February 15, 2018, the Yukon Government granted permission for the intervention to proceed for the remainder of the study period on the condition that the cancer warning label be rotated out permanently [66,67]. Based on remaining label stock, an estimated 47,000 cancer warning labels and 53,000 LRDG labels were applied to alcohol containers during the first month. On April 12, 2018, label application resumed in the intervention site, starting with the LRDG labels and followed by the standard drink labels on May 28, 2018; application continued until July 31, 2018 (Figure 7). An estimated 117,000 LRDG and 92,000 standard drink labels were applied during that period. Yukon’s original alcohol warning labels cautioning about drinking during pregnancy were not applied to containers at any point during the intervention period.

### Study Implementation

**Figure 8:**
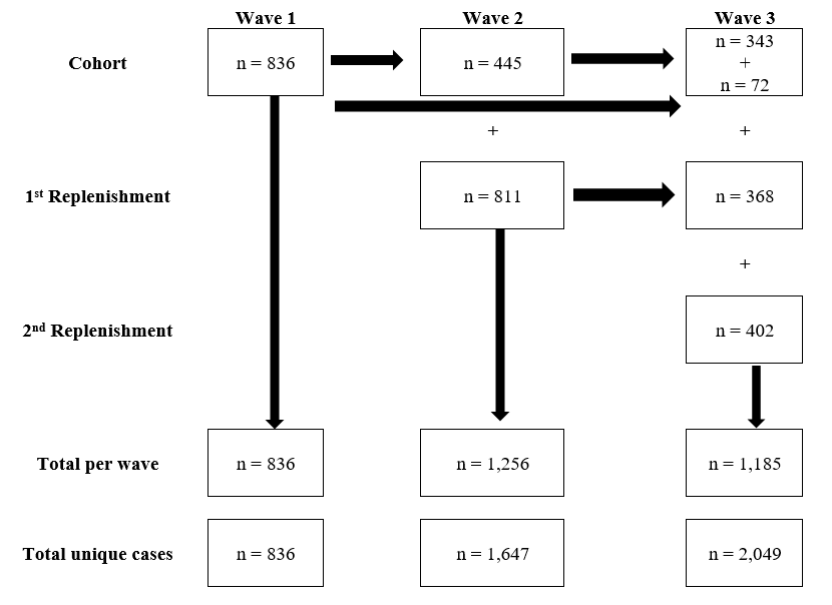
Structure of the survey cohort showing attrition and replenishment across waves.

The baseline (Wave 1) surveys were completed over a 6-week period in May and June 2017. The study team was granted permission to conduct an extra wave of surveys (Wave 2) in both the intervention and comparison sites for a 6-week period in February-March 2018 to capture any effect of the cancer label. The third and final wave of surveys (Wave 3) was conducted for 6 weeks at both sites between mid-June and the end of July 2018 (see Figure 8 for sample sizes across waves). In total, 2049 participants were eligible and completed a survey in at least one of the three survey waves. Overall, response rates were 8.9% at the intervention site and 8.0% at the comparison site [68], which were low but consistent with previously published studies using similar intercept techniques [69-71]. Cooperation rates of 97.6% at the intervention site and 95.5% at the comparison site were achieved across survey waves. Overall, 53.2% (445/836) participants were retained in Wave 2 and 47.5% (783/1647), in Wave 3. Participants who were lost to follow-up between waves were more likely to be younger; male; have lower education, income, and literacy; consume high or unknown levels of alcohol; and be at the comparison site (Multimedia Appendix 1). A number of components of the social marketing and awareness campaign were implemented during the intervention period. These components included an initial media release about the alcohol warning labels, a toll-free helpline number, and an informational website hosted by the Yukon Liquor Corporation that offered additional resources such as more detailed LRDG and standard drink information [72]. The Yukon Liquor Corporation was not able to implement the other planned elements of the campaign during the intervention period, including promotional materials such as a “drink counter” fridge-magnet notepad, in-store posters, and related radio spots by the Chief Medical Officer of Health.

## Discussion

### Principal Results

This study presented a rare opportunity to conduct a quasi-experiment with high internal and external validity and the potential to provide real-world evidence to directly inform alcohol labelling policies in Canada and internationally. Moreover, it provided a unique opportunity to collaborate with northern governments in Canada to test a population-level intervention among an understudied, high-priority northern population that has a higher prevalence of alcohol intake and alcohol-related harms relative to the rest of Canada [2]. This research was the first study in Canada and one of the few internationally to examine the effects of best-practice alcohol warning labels and determine if these labels are an effective population-level intervention for increasing awareness of alcohol-related harms as well as reducing alcohol consumption. Unimpeded, it had the potential to make a unique contribution to the evidence and provide critical information to develop and refine communication approaches and alcohol-labelling policies.

As found previously, there are difficulties associated with introducing or testing alcohol warning labels in real-world settings, largely as a result of alcohol industry interference or pushback and increasing reluctance from governments [19,20]. In this study, representatives from the national alcohol industry in Canada raised their objections to the alcohol warning labels intervention with government officials immediately following the launch. Records of email communication with the Yukon Liquor Corporation illustrate the industry representatives’ ongoing attempts to discredit the research team and distort the evidence linking alcohol and cancer [73]; a letter of complaint was sent to one of the study leads’ university administration (Multimedia Appendix 2); and freedom of information requests for all communications related to the study for the research team and the territorial liquor corporations in Yukon and Northwest Territories were submitted. The industry’s opposition is understandable: They fear that strong warning labels will shrink their market and erode profits. In this case, their stated concerns included legislative authority for applying labels to containers, trademark infringement, and defamation and damages related to warning labels affixed to “brand-owner products” without consent [65]. However, it quickly became clear that the cancer warning label, in particular, was eliciting the strong response, which is consistent with broader industry positions on this type of health messaging [17,65]. To enable the intervention to proceed, the government partner ultimately modified their participation in the study to avoid what was understood as a risk of a lawsuit (however groundless) against the jurisdiction if they continued to apply the cancer label [67,74]. The threat of litigation is a deterrence tactic similar to what has been used previously by the tobacco industry, often resulting in protracted and expensive cases [75].

After the study was halted in December 2017, the research team actively engaged with the media to document the alcohol industry’s attempts to stop the study and highlight the threats of litigation being directed against Yukon. The territorial government’s decision to resume participation in the study can largely be attributed to unwavering support from several prominent public health and government officials and academics both in Yukon and across Canada as well as wide public interest due to the national and international media coverage [74,76]. The research team’s partnership with the government-run liquor corporation that had developed over a number of years during the planning of the study also greatly facilitated the ability to expedite modifications to the study design in response to the unplanned interruption and maintain as much of the integrity of the original protocol as possible.

### Limitations

Although a significant limitation of the study is the postimplementation modifications that were required as a result of the alcohol industry’s attempts to stop the study and some related media coverage, findings from the subsequent waves of postintervention data will provide an indication of the extent to which the new enhanced alcohol warning labels impacted key outcomes. One benefit of the industry interference was that it provided detailed clarification on the potential industry response to enhanced alcohol warning labels and an example of how the intervention was modified to ensure successful completion of the study. Additional study limitations include participants being recruited using nonprobability methods, precluding the findings from being representative estimates of broader alcohol-consumer populations, and use of self-report surveys that may be subject to social-desirability bias. Analysis of liquor store sales data from both sites is a strength of this study and will provide objective evidence of changes in population-level alcohol consumption. Finally, some elements of the planned social marketing and awareness campaign were not launched during the intervention period; thus, the comprehensiveness of the educational strategy was not fully realized.

Implementation of stronger policies and safeguards to prevent alcohol industry interference with scientific scholarship are recommended, so that further unimpeded, real-world testing and implementation of enhanced, best-practice alcohol warning labels can proceed in the future. Incorporating proactive media engagement with messaging around potential industry responses to alcohol warning label initiatives as part of the research design may help protect future studies from industry interference.

## Appendix

Multimedia Appendix 1 Participant flow diagram across waves.

Multimedia Appendix 2 Letter of complaint from alcohol industry sent to the University of Victoria.

## Abbreviations

CPI: consumer price index
LRDG: low-risk drinking guidelines
