# Supplementary figures and images for: Testing the Effectiveness of Enhanced Alcohol Warning Labels and Modifications Resulting From Alcohol Industry Interference in Yukon, Canada: Protocol for a Quasi-Experimental Study
Source: JMIR Res Protoc. 2020 Jan 10;9(1):e16320. doi: 10.2196/16320 (PMC6996737; doi:10.2196/16320)

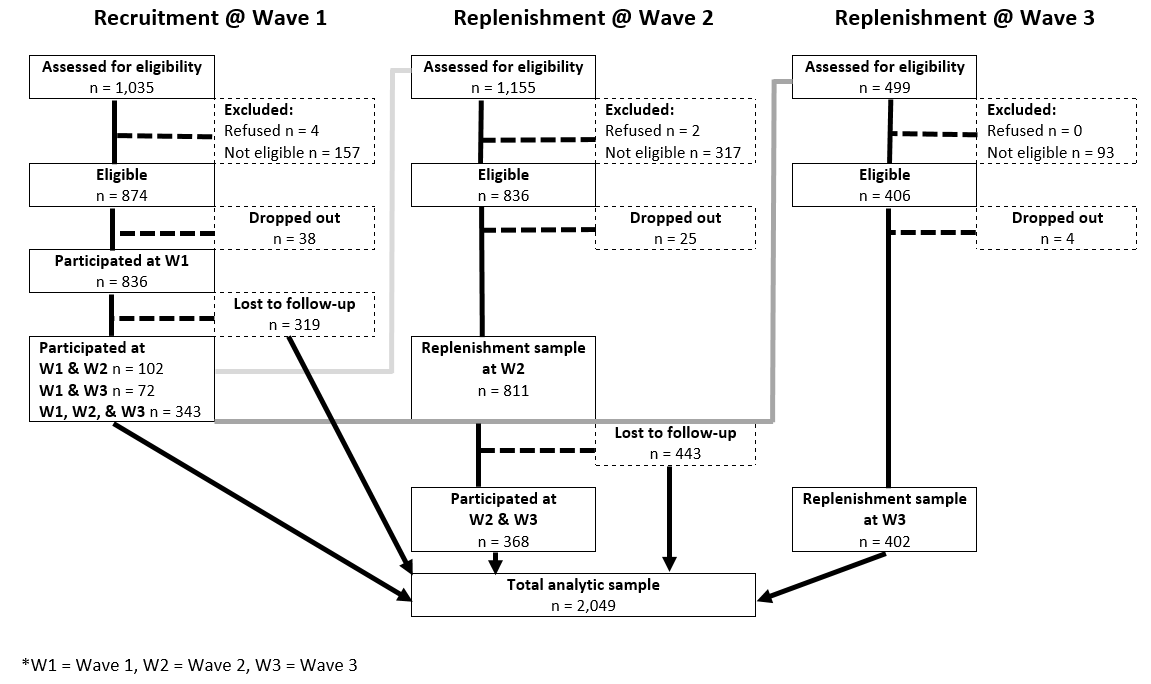

Supplement: Multimedia Appendix 1 [file resprot_v9i1e16320_app1.PNG]
