# Supplementary material for: Testing the Effectiveness of Enhanced Alcohol Warning Labels and Modifications Resulting From Alcohol Industry Interference in Yukon, Canada: Protocol for a Quasi-Experimental Study
Source: JMIR Res Protoc. 2020 Jan 10;9(1):e16320. doi: 10.2196/16320 (PMC6996737; doi:10.2196/16320)
Supplement: Multimedia Appendix 2 [file resprot_v9i1e16320_app2.pdf]

December 14,2017

Vice-President Research
University of Victoria
Room A110, Administrative Services Building,
3800 Finnerty Rd. (Ring Road)
Victoria BC V8P 5C2

Subject: Dr. Tim Stockwell – Yukon / NWT Northern Territories Alcohol Study – Ethics Board Approval Inquiry

I have learned through media reports that a project is being undertaken in the Yukon that involves surveying beverage alcohol consumers for awareness of alcohol warning labels being tested as part of pilot that feeds into a larger study. I have a significant interest in this project and in particular the nature of the pre-pilot survey structure and post-pilot follow-up. I am very concerned that the project has been designed to ensure an outcome that aligns with the researchers' agenda.

I am also concerned that the labels being piloted are false, misleading, conflicted and potentially dangerous. Here are my concerns with two of the three labels I have seen in the media:

- "Alcohol can cause cancer" is a false statement intended to mislead and alarm consumers.
- "2 drinks a day for women and three for men" might be intended to convey the Low Risk Drinking Guidelines but could be interpreted by consumers to mean the amount that is safe to drink and still drive. This is dangerous.

As the Executive Director of the British Columbia Craft Brewers Guild who represents over 100 independently owned and operated businesses I want to ensure this research will be fair and unbiased. As the Attorney General of BC Mr. Eby has spoken about this pilot, it makes it relevant to our craft beer community.

I would like to know if the project, where the University of Victoria's name is used and Dr. Tim Stockwell is one of the principal investigators and spokespeople, was cleared through University of Victoria's Ethics board. If it was, could I get a copy of the Board's review. If not, can you help me understand why it would not have gone through the Ethics Board.

Thank You

Ken Beattie
Executive Director – BC Craft Brewers Guild
Ken@bccraftbeer.com
